# Fertility intentions among HIV positive women aged 18–49 years in Addis Ababa Ethiopia: a cross sectional study

**DOI:** 10.1186/1742-4755-11-36

**Published:** 2014-05-20

**Authors:** Hussen Mekonnen Asfaw, Fikre Enquselassie Gashe

**Affiliations:** 1 Department of preventive medicine, School of Public Health, Addis Ababa University, Addis Ababa, Ethiopia

**Keywords:** Fertility intension, HIV, ART use, Women, Addis ababa, Ethiopia

## Abstract

**Background:**

Given the degree of HIV epidemic among women and the current antiretroviral therapy (ART) scale up in Ethiopia; considering the issue of fertility is vital to ensure the delivery of integrated reproductive health along with prevention services provided to positive women. This study was aimed to assess fertility intentions of women living with HIV attending public health institutions (hospitals & health centers) in Addis Ababa, Ethiopia.

**Methods:**

Institution based cross sectional survey was conducted, among 1855 HIV positive, women aged 18–49 years selected from different public health facilities in Addis Ababa; from June to October 2012. Information was gathered by using interviewer administered questionnaires. Data were double entered in EPI Info version 3.5.2 software, cleaned finally exported to IBM SPSS statistics version 20 for analysis. Logistic regression models were used to predict the association of study variables and adjusted for possible confounders.

**Result:**

Overall, 44% of women reported fertility intention. ART users had higher fertility intention **(AOR; 1.26, 95%CI; 1.01 to 1.60)** than ART naïve. In addition to this, having partner being on sexual relationship, young age, being single and having fewer or no children were found to be predictors of fertility intentions. The presence of ART, improvement of health condition and the influence of husband were the main reasons for childbearing intentions of women in the study area.

**Conclusion:**

A considerable proportion of women reported fertility intention. There was an association between fertility intentions and ART use. It is important for health care providers and policy makers to strengthen the fertility need of HIV positive women along with HIV care so that women may decide freely and responsibly on their fertility issues.

## Introduction

Human immune deficiency virus (HIV) continues to profoundly affect women across the world [1]. The burden is however, heaviest in sub-Saharan Africa [2]; approximately 58% of adults living with HIV [1] and 53% of all adult deaths in the region are women [3]. In Ethiopia women (2.9%) than men (1.9%) are living with HIV [4].

Most of these women are particularly vulnerable to HIV due to complex burden they have [5] including physiological, social vulnerability and gender inequalities [3]. Since infected women are of childbearing age [4], they risk infecting their children and thus face difficult choices about childbearing.

Despite the risks and challenges; studies elsewhere revealed that, HIV positive women continue to desire children, after knowing their HIV-positive status [6-8]. Even infection like HIV does not seem to negatively modify subsequent reproductive intentions of some patients [9,10]; also majority of them intended to have more than two children [11,12]. The desire of having children in the future has significant implication for the transmission of HIV to sexual partner and new-born [13,14].

The advent of antiretroviral therapy (ART) has improved quality of life and survival for people living with HIV(PLWH) and now days HIV infection can be seen as a chronic manageable disease as a result many will contemplate child bearing [5,15-17].

With access to ART and preventive care, people living with HIV are better able to consider parenthood [18] as compared to early days. Earlier studies involving women on ART have revealed that most of pregnancies among positive women might be intentional [19-21]. Subsequent studies from sub-Saharan Africa reported antiretroviral therapy access has an influence on fertility intentions of HIV positive women [10,22,23].

ART has not only transformed their physical state but it has also transformed mostly what had been desire into intention [24]. Since childbearing intentions are among predictors of subsequent fertility behaviors of women [25], hence creating responsive reproductive health services to HIV positive women in relation to ART, requires clear understanding of expressed childbearing intentions.

Although, ART has influence in improving quality of life and reduce mother to child transmission of HIV virus (MTCT) in developed countries [21]; vertical transmission accounts for more than 90% of pediatric AIDS in prevalent areas [26] as a result, reproductive assistance to HIV positive women [17,27] is important to have informed decision making about child bearing and child rearing [15].

Indeed existing evidences in Ethiopia have indicated fertility desire of positive men and women was ranging from about 34 to 45% [28-31]. However information on situation of fertility intentions, differences of fertility intentions by ART use and reasons to have future children among HIV positive women are lacking.

Ethiopia is a country characterized by high fertility 5.6% children per childbearing woman [4], cultural value is attached with children and being childless considered as social violation and stigma. While majority (91%) of women deliver at home without skilled attendant [32]; identification of factors associated to have children among HIV positive women is useful for establishing intervention priorities in reproductive health for these populations.

Furthermore, in the light of preventive efforts undergoing in Ethiopia, it becomes imperative to assess fertility intention, with emphasis to HIV positive women who have been enrolled in care; in order to proffer appropriate prevention measures and better integration of reproductive health services.

Thus, this study will attempt to update existed knowledge and inform the policy makers and programs to promote efforts for provision of safer and healthier reproductive options among HIV positive women in the study area.

## Methods

### Study area

The study was conducted in Addis Ababa, the capital city of Ethiopia and the seat of the African Union. As a chartered city, Addis Ababa has the status of both a city and a state. From its lowest point, around Bole International Airport, at 2,326 m above sea level in the southern periphery, the city rises to over 3,000 m in the Entoto mountains to the north [33,34]. Addis Ababa has 10 sub-cities (top layers) and 116 woredas (lower administration units). Each sub-city is expected to serve for a total population of 300,000 and each woreda serving for the population of 30,000 [34].

Fee based antiretroviral treatment began in 2003 and free ART was launched in Ethiopia in 2005 [35]. Both public and private health institutions have been offering ART services in the city [36]. A total of five public hospitals, twenty five health centers, one clinic and thirteen private hospitals were offering ART [37] and about 124,983 enrolled, 76,035 ever started and 54,667 currently on ART [4].

### Study design and sampling procedures

Institution based cross sectional survey was conducted in selected public health facilities (hospitals & health centers) of Addis Ababa, from June to October, 2012.

Sample size was determined using single proportion formula with the assumption of fertility desire of HIV positive women being 44.7% [30] with 5% level of significance a design effect of 2 and an addition of 10% non response rate. With these assumptions, the minimum required sample size was 806. However, the total sample size calculated for comparison purposes was 1924. As large sample size increases the precision of the findings by reducing the level of sampling error [38], we took 1924 HIV positive women aged 18–49 years.

Respondents were selected principally using multi-stage sampling technique. Initially, unique identification number was given to each public hospitals and health centers. Secondly, a total of five public hospitals and seven health centers were selected by lottery methods, then proportion of sample size allocations were carried out according the patient load in each public institution. Systematic random sampling was used to select the study subjects from the selected ART sites.

### Data collection

Data were collected using interviewer administered structured questionnaires. The data collection tool was adopted from previous similar studies [23,39] and adapted in to the local context. The data collection instrument was prepared in English and translated in to the local language (*Amharic*), by expert who is fluent in both languages and back translated to English by another expert to ensure consistency and accuracy.

Data collectors were recruited based on previous experience in data collection, relevance of qualification, training on (VCT, ART) and ability of the local language. Training was given for five consecutive days in order to make data collectors and supervisors familiar to the tool and interview techniques. Emphasis was also given during training on the ethical issues, safety of the participants as well as interviewers, minimization of under-reporting and maintaining confidentiality. The data collection tools were pre-tested in the randomly selected ART units out of the study site on 10% of the total sample size.

A field work manual was developed by the principal investigator and used by all research teams. To ensure the quality of the data and minimize inter-interviewer variation, about 5% of the respondents were re-interviewed at random by the principal investigator and supervisors and checked for consistency. In addition, daily cheek and follow up were done by the supervisors and investigator.

### Measurement

The primary outcome variable for the study was self-reported fertility intentions, which was defined by answers to the question: “Are you currently planning to have (more) children in the future?” Women were free to respond “Yes”, “No”, or “Don’t know, the small proportion of women who responded “Don’t Know” (less than 5%) were included in the “No” category. There was little difference in the proportion of women reporting “Don’t know” by HIV and HAART use status. Variables known to be associated with fertility intentions were included in the analysis to provide an adjusted estimate of the association.

Covariates included were age, religion, marital status education, employment, monthly household income, current sexual partnership status, number of living children, and HIV clinical variables including recent CD4 count, nadir CD4 count, and WHO stage of disease. Medical record review was also conducted to confirm HAART history and obtain clinical data, including World Health Organization stage of disease and CD4 cell count.

Antiretroviral therapy (ART) use was defined as use of one of three antiretroviral medications either efaveranze(EFV) or Neverapin(NVP) based [5,35,39], first line drugs or use of non nucleotide reverse transcriptase inhibitors (NRTIs) with protease inhibitors(PIs) (leponavir/ritonavir(LPV/r) or atazanavir/ritonavir (ATV/r)) backbone of second line drugs [35].

### Analysis

The pre coded responses were double entered in EPI Info version 3.5.2 software, for checking its consistency then was exported to SPSS for window version 20 for statistical analysis used were percentage, frequency, bivariate and multiple logistic regression analysis. Variables found to be significant at bivariate level, (P < 0.05), were selected and included in to multiple logistic regression models. Then multiple logistic regression analyses model were used to calculate Odds ratio with 95% confidence interval to estimate association and to control the potential confounding variables. Strength and direction of the association presented using odds ratios relative to the reference category and 95% confidence levels.

### Ethical considerations

The research was approved for scientific and ethical integrity by the Institutional Review Board (IRB) of College of Health Sciences, Addis Ababa University. Written permission was obtained from health bureau of the Addis Ababa city government. Consent was obtained from medical directors and respective unit heads at each health institutions. Verbal consent was also obtained from individual clients. In order to make informed decision, sufficient information was given to each participant. Confidentiality was strictly maintained for each piece of information and the interview was conducted in strict private place. At the end of the interview general information, referral and follow up linkages were made for those who need.

## Results

### Socio-demographic characteristics of study participants

A total of 1924 women were approached for participation (Table 1), of whom 1855 (96.4%) consented to participate in the study. Five hundred forty six (29.4%) were in the age range of 30–34 years. the mean age of the respondents was 31.3 years (±5.6SD).

**Table 1:** Socio-demographic characteristics of HIV positive women aged 18–49 years in Addis Ababa, Ethiopia (n = 1855)

**Characteristics**	**Frequency (%)**
**Age(years)**	
18-24	189 (10.2)
25-29	522 (28.1)
30-34	546 (29.4)
35-39	421 (22.7)
40-49	177 (9.5)
**Mean age in years**	**31.3 (±5.7SD)**
**Ethnicity**	
Amhara	1104 (59.5)
Oromo	420 (22.6)
Gurage	165 (8.9)
Others(Tigrea, Siltea, Gamo, welayta, yem, worji)	166 (8.9)
**Religion**	
Orthodox Christian	1461 (78.8)
Muslim	197 (10.6)
Others (Protestant, catholic, Jubbah)	197 (10.6)
**Marital Status**	
Single	361 (19.5)
Married/cohabited	945 (50.9)
Widowed	283 (15.3)
Divorced	266 (14.3)
**Educational status**	
Illiterate	243 (13.1)
Informal education	150 (8.1)
Grade 1–8 completed	689 (37.1)
Grade 9–12 completed	599 (32.3)
Above 12 grade	174 (9.4)
**Occupation**	
Unemployed	379 (20.5)
Housewife	342 (18.4)
Daily laborer	279 (15.0)
Merchant	100 (5.4)
CSW**	34 (1.8)
Government worker	212 (11.4)
Private Business	509 (27.4)
Monthly income (in Birr)*	
No Income(dependent)	522 (28.1)
Birr below 500	509 (27.4)
Birr 500-3000	521 (28.1)
Birr 1001-3000	258 (13.9)
Birr above 3000	45(2.4)

*1USD 18 Birr (currency of Ethiopia at the time of data collection), **CSWs commercial sex workers.

The study participants were predominantly Orthodox 1461 (78.8%) and Amhara 1104 (59.5%) by their religion and ethnicity respectively. Three hundred seventy nine (27.4%) were unemployed and 509 (20.5%) reported working in private business. Six hundred eighty nine (37.1%) of the participants have completed grade 1–8, followed by those who completed grade nine to twelve 599 (32.3%). Five hundred twenty two (28.1%) reported to have no monthly income at all and 509 (27.4%) were getting monthly income of less than 500 Ethiopian Birr (equivalent to 27.8 USD). Concerning marital status about 50% of the participants were currently married and 19.5% were single.

### Fertility intentions and clinical characteristics of study participants

Eight hundred fifteen (44%) women reported that they intended to have (more) children in the future irrespective of their ART use (Table 2).One thousand sixty two (57.3%) women reported ever had sexual relationship. Eight hundred sixty four (81%) women reported that their sexual partner/husband being tested for HIV of whom 630 (73%) reported that their partners are positive; while 217 (25%) reported that their partners were negative. Six hundred and five (32.6%) women reported having one child followed by 408 (22.0%) two children and 558 (30.1%) no children.

**Table 2:** Fertility intention and clinical characteristics of HIV positive women aged 18–49 years in Addis Ababa, Ethiopia (n = 1855)

**Fertility intention and clinical characteristics**	**Freq. (n =%)**	**Fertility intention and clinical characteristics**	**Freq. (n =%)**
		**Recent CD4 count **(n = 1855)	
**ART use** (n = 1855)		<200	268 (14)
**Yes**	823 (44.3)	200 to <350	589 (32)
**No**	1032 (55.7)	350 or greater	998(54)
**Fertility intention** (n = 1855)		**Nadir CD4 Count (Lowest)** (n = 1855)	
**Yes**	815 (44)	Less than 200 cell/mm^2^	785 (42)
**No**	1040 (56)	200 to less than 350 cell/mm^2^	810 (33)
**Have sexual partner** (n = 1855)		350 and above	460 (25)
Yes	1062 (57.3)	**WHO stage of disease** (n = 1855)	
No	793 (42.7)	Stage I/II	1096 (59)
**Partner tested** (n = 1062)		Stage III/IV	759 (41)
Yes	864 (81)	**Disclosed HIV Status to husband/partner** (n = 1062)	
No	104 (10)	Yes	906 (85)
Do not know	94 (9)	No	140 (13)
**HIV status of partner** (n = 864)		No response	16 (2)
Positive	630 (73)	**Disclosed HIV status to someone else **(n = 1855)	
Negative	217 (25)	Yes	1655 (89.2)
Do not know the status	17 (2)	No	200 (10.8)
**Ever lost a child by HIV **(n = 1297)		**Number of living children **(n = 1855)	
Yes	45 (3)	0	558 (30.1)
No	1228 (95)	1	605 (32.6)
Do not know the cause	24 (2)	2	408 (22.0)
		3+	284 (15.3)

One thousand six hundred fifty five (89.2%) women have disclosed their sero-status to someone else, but the remaining 200 (10.8%) did not. Of those who disclosed, 906 (85%) reported the disclosure of their sero-status to sexual partner/husband, but, 140 (13%) did not disclose their sero-status to their sexual partner.

### Factors associated with fertility intentions

ART users had higher fertility intention **(AOR; 1.26, 95% CI; 1.01 to 1.60)** than HAART naïve. Women in the age range of 40–49 years **(AOR; 0.14, 95% CI, 0.08 to 0.24**), age range 35–39 **(AOR, 0.46; 95% CI, 0.31 to 0.66)** and age range 30–34 **(AOR, 0.60; 95% CI, 0.42 to 0.84**) were respectively less likely to report fertility intention in comparison to younger women in the age range of 18–24 years (Table 3).

**Table 3:** Adjusted analysis of variables associated with fertility intentions among positive women aged 18–49 years in Addis Ababa (n = 1855)

**Variables**	**Fertility intension**		**Odds ratio (95% CI)**
	**Yes**	**No**	**AOR (95% CI)**
**Age ( years)**	N (%)	N (%)	
18-24	112 (13.7)	77 (7.4)	1.00
25-29	284 (34.8)	238 (22.8)	0.81 (0.57-1.15)
30-34	245 (30.1)	301 (28.9)	0.60 (0.42-0.84)*
35-39	151 (18.5)	270 (26)	0.46 (0.31-0.66)
40-49	23 (2.8)	154 (14.8)	0.14 (0.08-0.24)
**Marital status**			
Single	190 (23.3)	171 (16.4)	1.00
Married/cohabited	485 (59.5	460 (44.2)	1.10 (0.34-1.41)
Widowed	47 (5.8)	236 (22.7)	0.26 (0.18-0.39)
Divorced	93 (11.4)	173 (16.6)	0.59 (0.42-0.83)
**Educational status**			
Illiterate	72 (8.8)	171 (16.4)	1.00
Informal education	57 (7.0)	93 (8.9.0)	1.43 (0.91-2.27)
Grade 1–8 completed	306 (37.5)	383 (36.8)	1.62 (1.15-2.27)*
Grade 9–12 completed	286 (35.1)	313 (36.8)	1.74 (1.21-2.50)
Above grade 12	94 (11.5)	80 (7.7)	2.16 (1.32-3.53)
Currently having partner/husband			
No	257 (31.5)	536 (51.5)	1.00
Yes	558 (68.5)	504 (48.5)	0.46 (0.25-0.84)
**Number of living children**			
0	384 (47.1)	174 (16.7)	1.00
1	301 (36.9)	304 (29.2)	0.34 (0.26-0.44)**
2	95 (11.7)	313 (30.1)	0.08 (0.06-0.11)
3+	35 (4.3)	249 (23.9)	0.30 (0.02-0.06)
**ART use**			
ART- naïve	339 (41.6)	484 (46.5)	1.00
Receiving ART	476 (54.4)	556 (53.5)	1.26 (1.01-1.60)**
**Partner tested**^***#***^			
*Yes*	466 (44)	398 (37)	1.00
*No*	53 (5)	51 (5)	0.8 (0.6-1.3)
*I do not know*	39 (4)	55 (5)	0.6 (0.4-0.9)*

**#**considers only those who have reported presence of partner =1062 * P value <0.05, **P value <0.001.

Marital status has also an association with fertility intension in with divorced were (AOR, 0.59 95% CI, 0.42 to 0.83) and widowed women (AOR; 0.26; 95% CI; 0.18 to 0.39), respectively had less fertility intention to that of single women. However, no significant association with fertility intention was found between married/cohabited and single women (AOR; 1.10, 95% CI; 0.34 to 1.41). Women who completed grade eight (AOR, 1.62, 95% CI; 1.15 to 2.27) and those who completed grade 12 and above were two times (AOR; 2.16; 95% CI; 1.32 to 3.53), respectively, were more likely to have future children than illiterate women.

Fertility intention also declines by number of children women have. The odds of fertility intention for women who have one child (AOR, 0.34, 95% CI, 0.26 to 0.44), women who have two children (AOR; 0.08, 95% CI, 0.06 to 0.11) and women with three or more children (AOR: 0.30, 95% CI; 0.02 to 0.06) than women with no children, respectively. HIV test of the partner has an association with fertility intention, women who do not know whether their partner have taken HIV test or not were less likely to report fertility intention (AOR, 0.44; 95% CI; 0.25 to 0.78) than women who knew the test of their partner, while women who are living with partner who did not test at all were equally likely to report fertility intention. The finding also reviled that women who have sexual relationship were less likely to report fertility intention (AOR, 0.46, 95% CI, 0.25 to 0.48) than women who did not report presence of sexual relationship with partners (Table 3).

### Reasons to have children

**Figure 1:**
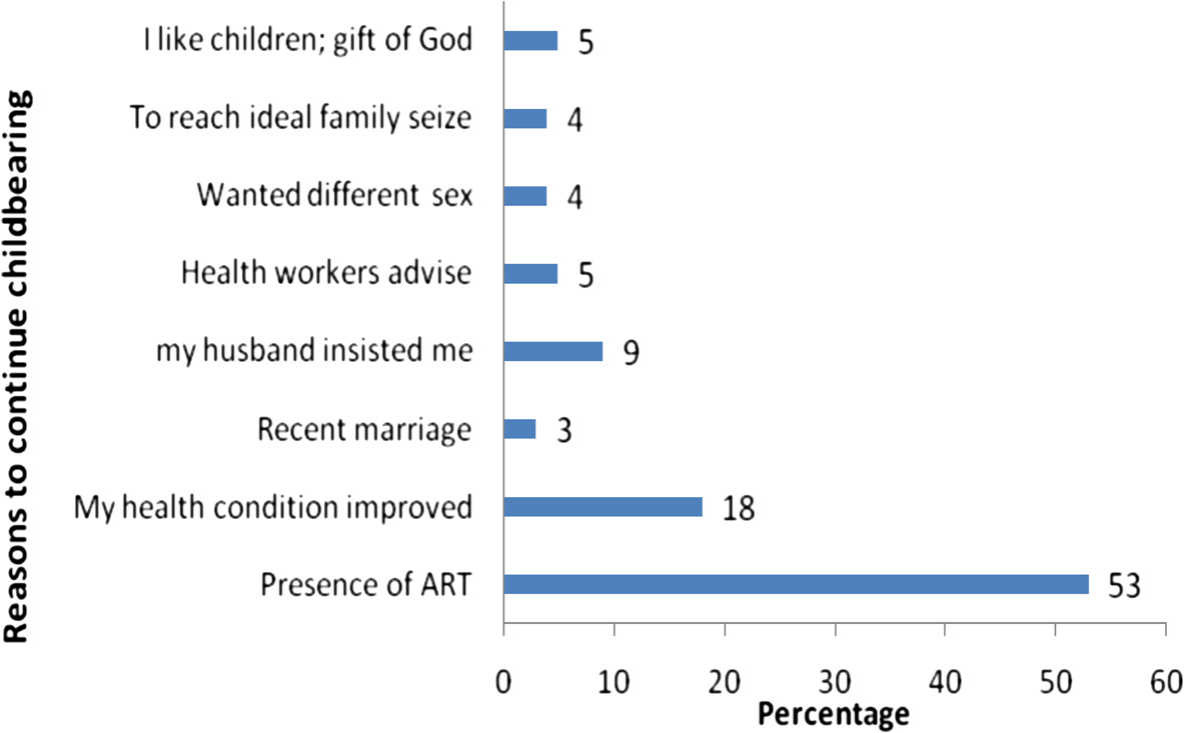
Reasons to have children in the future among positive women, aged 18-49 years in Addis Ababa (n=815).

There was a wide range of reasons why women intended to have additional children as shown in (Figure 1). Four hundred thirty seven (53%) women reported as a result of the presence of ART, followed by improvement of health condition 145 (18%). Other reasons mentioned were influence of husband/partner 70 (9%), like children as they are gift of God 41 (5%), advice of health workers 37 (4%), to reach ideal family size 34 (4%) and the like.

## Discussion

Our finding highlighted that the prevalence of fertility intention is 44% which is consistent with the previous studies in South Africa 44% & 45% respectively [22,23]; but higher than reports from northern part of the country (18.5%) [40], Uganda (24% and 28.6%) respectively [17,41], Canada (25.8%) [21] and lower than other study from Canada( 57%) [42]. The difference might be related to study site, time and study subject difference. The other plausible difference could be associated that being HIV positive did not remove childbearing intentions rather there exist diversity.

The proportion of women who reported fertility intention also showed variation with being on ART; this might be an indication of women hoping the presence of ART. Furthermore, wide range of reasons were reported as to why women intended to have children; such as presence of ART, improvement of health conditions, influence of husband, like children as they are gift of God, advice of health workers and to attain ideal family size. This suggests the need of integrating target oriented and individualized counseling along with comprehensive care and support activities. This study has shown that HAART use has an association with fertility intentions in which women who are on HAART reported more fertility intention over those who did not. The finding is consistent with previous studies [11,43] but different from other studies in other Uganda and South Africa [17,23]. The difference might be associated with variation in terms of ART access and community awareness. The other plausible reason might be related to multifaceted nature on the predictors of childbearing intentions. Our finding also indicated that no association with duration of ART and fertility intention which is similar to previous study from South Africa [23].

The study has indicated that women being single, low parity and young age were directly associated with fertility intention, the finding is similar to study conducted elsewhere in south Africa [23] but different from studies conducted in Uganda and Zimbabwe [17,24]. The difference is related to study subject difference.

As it is indicated those widowed and divorced were less likely to report childbearing intention compared to single. The probable reason might be related to those groups of women had children or social instability might impose fear about childbearing. However, single women might intend children from cultural influence; in Ethiopian community having children is highly valued [40] and considered as social security.

The respondents with twelve grades and above had two (AOR 2.16)-fold higher in childbearing intention compared to illiterates, this is similar to study conducted in South Africa [23]. This is related to participants who under gone formal education might hope the presence of ART. Furthermore, education especially of women, contributes to decision-making autonomy on fertility issues and knowledge of family planning. Studies have shown that women’s relative decision-making autonomy, independent of men authority facilitates fertility regulation [44,45].

Overall, being on ART, young age, being single, having fewer or no children, education of grade twelve and above, were identified to be predictors of fertility intensions. However, the sero-status result of spouse, religion, ethnicity, income, occupation and clinical profile such as WHO staging, current CD4 and nadir CD4 cell count respectively were not predictors of fertility intention among HIV positive women in the study area.

### Limitations

Our study has several limitations; the cross-sectional nature of the study may cause difficulty of determining the direction of the association between study variables and the association can only be discussed in terms of plausibility. There was a risk of social desirability bias where by HIV-positive women may over or under report their fertility intension because of pressure from health workers and community members to practice protected sex.

As to the strengths of this study, the respondents have been selected by random sampling technique with relatively large sample size. Again the team already adopted instrument conducted in other developing countries [23]. Precautions have been taken during the selection of experienced data collectors.

## Conclusion

Our finding suggests there is an association between fertility intentions and ART use, young age, being single, having fewer or no children in the study area. It is necessary integrated sexual and reproductive health services and HIV and ART care be available for HIV positive women. It is also important for health care providers and policy makers to strengthen the fertility need of HIV positive women along with HIV care so that women may decide freely and responsibly on their fertility issues.

## Competing interests

We declare that no financial or non-financial competing interests related to this study.

## Authors’ contributions

Both authors contributed equally during design and conduct of the study. HM and FE participated in data collection, statistical analysis and interpretation of findings. HM prepared the draft then FE revised the draft of the paper. All authors read and approved the final content of the manuscript.
